# Profiling Transcriptional Regulation and Functional Roles of *Schistosoma mansoni* c-Jun N-Terminal Kinase

**DOI:** 10.3389/fgene.2019.01036

**Published:** 2019-10-18

**Authors:** Sandra Grossi Gava, Naiara Clemente Tavares, Franco Harald Falcone, Guilherme Oliveira, Marina Moraes Mourão

**Affiliations:** ^1^Laboratório de Helmintologia e Malacologia Médica, Instituto René Rachou, Fundação Oswaldo Cruz, Belo Horizonte, Brazil; ^2^Allergy and Infectious Diseases Laboratory, Division of Molecular Therapeutics and Formulation, School of Pharmacy, University of Nottingham, Nottingham, United Kingdom; ^3^Institute of Parasitology, BFS, Justus Liebig University, Giessen, Germany; ^4^Environmental Genomics Group, Instituto Tecnológico Vale, Belém, Brazil

**Keywords:** *Schistosoma mansoni*, mitogen-activated protein kinases, c-Jun N-terminal kinase, RNA interference, signaling pathways, gene expression

## Abstract

Mitogen-activated protein kinases (MAPKs) play a regulatory role and influence various biological activities, such as cell proliferation, differentiation, and survival. Our group has demonstrated through functional studies that *Schistosoma mansoni* c-Jun N-terminal kinase (SmJNK) MAPK is involved in the parasite’s development, reproduction, and survival. SmJNK can, therefore, be considered a potential target for the development of new drugs. Considering the importance of SmJNK in *S. mansoni* maturation, we aimed at understanding of SmJNK regulated signaling pathways in the parasite, correlating expression data with *S. mansoni* development. To better understand the role of SmJNK in *S. mansoni* intravertebrate host life stages, RNA interference knockdown was performed in adult worms and in schistosomula larval stage. SmJNK knocked-down in adult worms showed a decrease in oviposition and no significant alteration in their movement. RNASeq libraries of SmJNK knockdown schistosomula were sequenced. A total of 495 differentially expressed genes were observed in the SmJNK knockdown parasites, of which 373 were down-regulated and 122 up-regulated. Among the down-regulated genes, we found transcripts related to protein folding, purine nucleotide metabolism, the structural composition of ribosomes and cytoskeleton. Genes coding for proteins that bind to nucleic acids and proteins involved in the phagosome and spliceosome pathways were enriched. Additionally, we found that SmJNK and Smp38 MAPK signaling pathways converge regulating the expression of a large set of genes. *C. elegans* orthologous genes were enriched for genes related to sterility and oocyte maturation, corroborating the observed phenotype alteration. This work allowed an in-depth analysis of the SmJNK signaling pathway, elucidating gene targets of regulation and functional roles of this critical kinase for parasite maturation.

## Introduction

Schistosomiasis is one of the most common human parasitic diseases whose socioeconomic impact affects people in developing nations in the tropics and sub-tropics. Preventive chemotherapy (PC) with Praziquantel is required in 52 countries, where only in 2017, 220 million people needed PC for schistosomiasis; however, only 44.9% of this demand was covered (WHO, 2018). Despite the control efforts, schistosomiasis continues to be a major public health problem, principally due to schistosomiasis-related morbidity (French et al., 2018; Nascimento et al., 2019).

*Schistosoma mansoni* kinome containing 252 eukaryotic kinase proteins (ePKs) was first described by Andrade and co-authors (Andrade et al., 2011) based on an earlier version of *S. mansoni* genome (Berriman et al., 2009). Recently, 351 kinase genes with evidence of being transcribed in nearly all adult stages were described, of which 268 were PKs and an additional 83 were non-PKs (Grevelding et al., 2017). Although protein kinases have been recognized for years as suitable targets for drug development (Cohen, 2002; Cai et al., 2017), experimental functional evidence exists for only 40 *S. mansoni* proteins, showing that there is still need for further research.

The c-Jun N-terminal kinase signaling pathway is involved in the developmental regulation of various organisms. Its role has already been demonstrated in oocyte maturation and embryogenesis of *Xenopus laevis* (Bagowski et al., 2001) and in the spindle assembly during the mouse oocyte meiotic maturation (Huang et al., 2011). In *S. mansoni*, SmJNK (Smp_172240) was previously characterized, demonstrating its importance for the establishment of infection in the mammalian host (Andrade et al., 2014). SmJNK knockdown caused damage to the adult worms’ tegument and impaired the maturation of vitelline organs. This resulted in lower oviposition and greater susceptibility to the host immune system. Furthermore, the SmJNK ortholog in *Schistosoma haematobium* was pointed as one of the prioritized druggable kinase targets due to its essentiality based on lethal gene knock-down or knock-out phenotypes in other organisms (Stroehlein et al., 2015).

Here, we sought to assess the role of SmJNK in adult worms *in vitro*, further advancing in unraveling the functions of this critical protein and putative druggable target. Also, we elucidated genes that are target of regulation by SmJNK knockdown, aiming to identify novel parasite-specific targets in addition to contributing to a better understanding of this signaling pathway. Additionally, we checked for a crosstalk between SmJNK pathway and the previously reported data from Smp38 (Avelar et al., 2019). Finally, we identified specific targets that correlate with previously observed phenotypes of developmental impairment (Andrade et al., 2014; Avelar et al., 2019) which is highly desirable for the design of new anti-schistosome drugs.

## Methods

### Parasites

*S. mansoni* adult worms of LE strain were recovered from hamster periportal perfusion 40 days after cercariae percutaneous infection (Pellegrino and Siqueira, 1956). Schistosomula were obtained by mechanical transformation of cercariae as previously described (Milligan and Jolly, 2011). Cercariae were supplied by the Mollusk Room ‘Lobato Paraense’ of the René Rachou Institute–FIOCRUZ, where the parasite cycle is routinely maintained. The sporocysts were prepared following the protocol previously described (Mourão et al., 2009). This work was approved by the Oswaldo Cruz Foundation’s Ethics Committee for Animal Use (CEUA) under number LW12/16, according to the Brazilian national guidelines set out in Law 11794/08.

### DsRNA Synthesis

For the dsRNA synthesis, an SmJNK mRNA fragment corresponding to a region of approximately 570 bp, previously cloned into pGEM-T Easy vector, was amplified by PCR. Primers and cycling conditions used in this study were previously designed and established by Andrade and collaborators (Andrade et al., 2014). After amplification, PCR products were separated on 1% agarose gels, purified using QIAquick Gel Extraction Kit (Qiagen) and used as the template for dsRNA synthesis. DsRNA synthesis was performed using the T7 RiboMAX Express RNAi Systems kit (Promega) according to the supplier’s protocol. DsRNA integrity and annealing were verified on 1% agarose electrophoresis.

### SmJNK Knockdown in Adult Worms by RNA Interference

After perfusion, males and females adult worms were washed and separated manually. Then, eight males and eight females were placed separately in each well containing 100 μL of RPMI 1640 medium with 25 μg of dsRNA, in two technical replicates. The worms were electroporated with specific SmJNK dsRNA or unspecific GFP dsRNA into 4 mm cuvettes at 125 V for 20 ms and cultivated in 24-well plates with 1 mL RPMI 1640 medium supplemented with 10% heat-inactivated Fetal Bovine Serum and 2% Penicillin/Streptomycin. Unless stated otherwise, all culture reagents were from Gibco, Thermo Fisher Scientific. Worm motility was assessed using the WormAssay software (Marcellino et al., 2012) for 10 days, in which eight worms were cultured in 24-well tissue culture plates containing 1 mL of medium. Similarly, to count the number of eggs laid, eight worm pairs were electroporated and cultured in 6-well plates, the medium was changed daily, and the eggs counted. Also, on days 3, 5, 7, and 10 after electroporation, two worm pairs per day were removed and macerated with TRIzol Reagent (Thermo Fisher Scientific) for RNA extraction as described below. SmJNK expression in adult worms was assessed by quantitative real-time PCR (RT-qPCR). Experiments were performed in three biological replicates. Data were analyzed using unpaired t-test with Welch’s correction.

### SmJNK Knockdown in Schistosomula by RNA Interference

Schistosomula cultures (∼500,000 worms/condition) were maintained in bottles with 10 mL of Glasgow Minimum Essential Medium (GMEM) (Sigma-Aldrich) supplemented with 0.2 µM triiodothyronine (Sigma-Aldrich); 0.1% glucose; 0.1% lactalbumin (Sigma-Aldrich); 20 mM HEPES; 0.5% MEM vitamin solution (Gibco, Thermo Fisher Scientific); 5% Schneider’s Insect Medium (Sigma-Aldrich); 0.5 µM Hypoxanthine (Sigma-Aldrich), 1 µM hydrocortisone (Sigma-Aldrich), 1% Penicillin/Streptomycin (Gibco, Thermo Fisher Scientific), and 2% heat-inactivated Fetal Bovine Serum (Gibco, Thermo Fisher Scientific). SmJNK dsRNA was added shortly after schistosomula transformation to a final concentration of 100 nM. Cultures were kept in a Bio-Oxygen Demand incubator (B.O.D.) at 37°C, 5% CO_2_, and 95% humidity. Two independent biological replicates were performed. Data were analyzed using unpaired t-test with Welch’s correction.

### Total RNA Extraction

RNA extraction was performed three, five, seven, and ten days after adult worm electroporation with SmJNK dsRNA. For schistosomula, the RNA extraction was performed two days after exposure to SmJNK dsRNA, as previously shown, reduction in transcript levels was greater at this exposure time (Andrade et al., 2014). For RNA extraction, TRIzol Reagent (Invitrogen) was used associated with the RNeasy Mini Kit (Qiagen) according to the manufacturer’s guidelines. Samples were treated with Turbo DNase (Ambion, Thermo Fisher Scientific) for removal of genomic DNA. RNAs were quantified using a Qubit Fluorometer 2.0 (Invitrogen) and, for RNASeq, RNA integrity was assessed using the Agilent RNA 6000 Pico kit and BioAnalyzer 2100 (Agilent Technologies).

### Gene Expression Analysis by Quantitative Real-Time PCR (RT-qPCR)

The cDNA was synthesized using SuperScript™ III Reverse Transcriptase (Invitrogen, Thermo Fisher Scientific) or Illustra Ready-To-Go RT-PCR Beads (GE Healthcare). qPCR was performed using Power SYBR^®^ Green Master mix (Applied Biosystems, Thermo Fisher Scientific) on an ABI 7500 RT-PCR system (Applied Biosystems, Thermo Fisher Scientific) to evaluate SmJNK knockdown. Primers and cycling conditions used in this study were previously designed and established by Andrade and collaborators (Andrade et al., 2014). Cytochrome C oxidase I gene (Smp_900000) was used to normalize RNA input from schistosomula (Andrade et al., 2014). FAD dependent oxidoreductase domain containing protein (SmFAD, Smp_089880) and actin-related protein 10 (Sm-arp 10, Smp_093230) genes were used to normalize RNA input from adult worms (**Supplementary Table S1**). In each plate, two internal controls were included to assess both reagent and genomic DNA contamination (RNA samples). Post-RNAi, SmJNK transcript levels were analyzed using the relative 2^-ΔΔCt^ method (Livak and Schmittgen, 2001) and expressed as the percentage of difference compared to the untreated or unspecific control.

To evaluate SmJNK expression among the different stages of *S. mansoni* we used qPCR absolute quantification using copy number standards, employing a curve containing five points of an SmJNK clone 10-fold dilution. The copy number of each dilution was calculated by the ratio of the clone molecular mass to the Avogadro constant as described (Lee et al., 2006), and the absolute copy number of the SmJNK transcript at each stage was estimated by interpolation of the sample Ct using a standard curve, and expressed as copy number per ng of total RNA.

### High-Throughput mRNA Sequencing (RNASeq)

After confirming SmJNK knockdown, 2 µg of total RNA extracted from untreated and SmJNK knocked-down schistosomula, two days after dsRNA exposure, were subjected to mRNA purification, fragmentation, cDNA synthesis, and library construction. Next generation barcode multiplexed sequencing libraries were constructed using the TruSeq stranded mRNA kit (Illumina) and pooled in equimolar concentrations. Libraries were sequenced as 100-base paired-end reads using HiSeq 2500 (Illumina) using a HiSeq Rapid SBS sequencing kit v.2 (Illumina). Raw sequence data were preprocessed using the standard Illumina pipeline to segregate multiplexed reads. Sequence quality was assessed using the FastQC program (http://www.bioinformatics.babraham.ac.uk/projects/fastqc). Reads were then mapped to the *S. mansoni* reference genome (v. 7) using the STAR program (v. 2.6.1d) using default parameters except the following: –outSJfilterReads Unique, –sjdbOverhang 99, –outFilterType BySJout, –outFilterMultimapNmax 20, –alignSJoverhangMin 8, –alignIntronMin 25, and –outSAMattributes All (Dobin et al., 2013).

### Screening of Differentially Expressed Genes

Downstream analysis was carried out using the R statistical software package (v. 3.3.2) (R Development Core Team, 2011). To ascertain genes that significantly change after SmJNK dsRNA exposure, we used DESeq2 (v. 1.12.4) (Love et al., 2014) to normalize the data and performed statistical analysis. Genes with adjusted p-value <0.01 were considered significantly differentially expressed. Clustering analysis was performed using the heatmap function from the gplots (v. 3.0.1) package and PCA plots were generated using the ggplot2 (v. 2.1.1) package.

Gene ontology (GO) (Ashburner et al., 2000; The Gene Ontology Consortium, 2017) and the Kyoto Encyclopedia of Genes and Genomes (KEGG) (Kanehisa and Goto, 2000; Kanehisa et al., 2016; Kanehisa et al., 2017) pathway analysis were used to identify the enriched molecular functions and associated biological pathways of differentially expressed genes (DEGs). The g:Profiler tool (version e95_eg42_p13_f6e58b9) (Reimand et al., 2007; Raudvere et al., 2019) was applied to perform the GO enrichment and KEGG pathway analysis. The g:SCS method was applied for computing multiple testing correction using the padjusted values with a 0.05 threshold. The ReViGO tool (Supek et al., 2011) was used to cluster, summarize, and visualize the list of significantly enriched GO terms based on their semantic similarities (Allowed similarity: 0.4).

We also compared DEGs of schistosomula SmJNK knocked-down with those found in schistosomula Smp38 knocked-down (Avelar et al., 2019). The down-regulated DEGs that overlap in both datasets were subjected to the BioMart tool (https://parasite.wormbase.org/info/Tools/biomart.html) from WormBase ParaSite (Howe et al., 2017) to search for orthologs in the nematode Caenorhabditis elegans. The orthologous genes were then analyzed using the Worm Enrichr enrichment analysis tool for *C. elegans* (https://amp.pharm.mssm.edu/WormEnrichr/) to search for the RNAi phenotypes associated with this gene list (Chen et al., 2013; Kuleshov et al., 2016).

### Quantitative Real-Time PCR Validation of Differentially Expressed Genes

Quantitative PCR was performed to validate the expression levels of selected differentially expressed genes. Ten genes were selected randomly; whereas five of them are among the DEGs exhibiting large fold changes (**Supplementary Table S1**). Specific primers were designed using PrimerQuest tool (https://www.idtdna.com/Primerquest/Home/Index) and/or Primer3 software (http://primer3.ut.ee/) and obtained from Integrated DNA Technologies (IDT). The sequences of all primers and the final concentrations established for each qPCR reaction are presented in **Supplementary Table S1**. PCR efficiency for each pair of specific primers was estimated by titration analysis to be 100% ± 10%. RNA extraction, purification, cDNA synthesis, and RT-qPCR reactions were carried as described above. SmFAD and Sm-arp 10 genes were used to normalize RNA input (**Supplementary Table S1**).

## Results

### SmJNK Expression Levels Among *S. Mansoni* Developmental Stages

**Figure 1:**
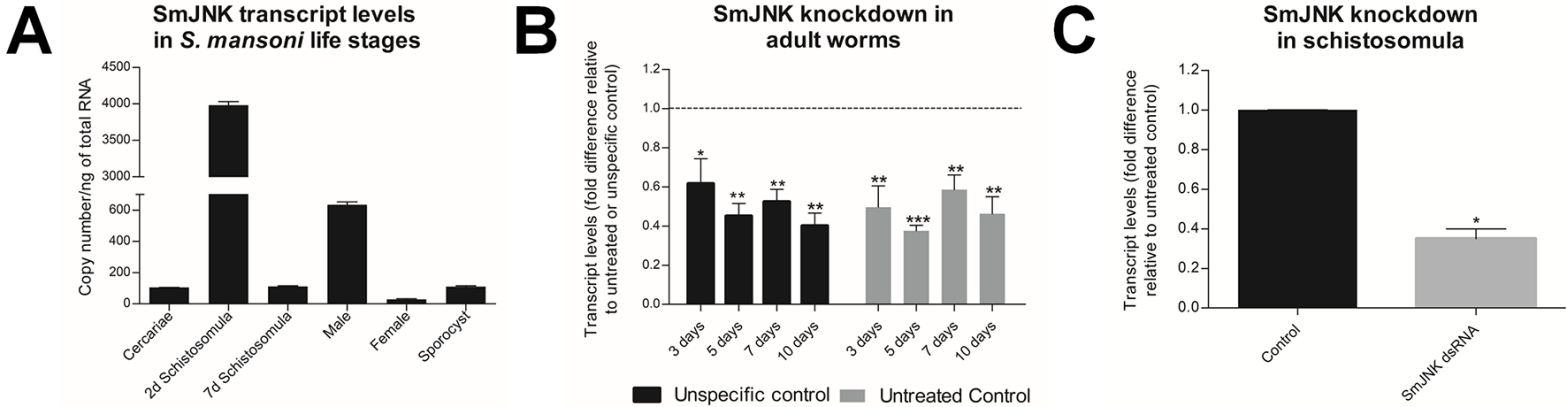
SmJNK expression profile among developmental stages of *S. mansoni* and after RNAi of adult worms *in vitro*. **(A)** SmJNK transcript levels in the different *S. mansoni* life stages; cercariae, two and seven days schistosomula, males, females, and sporocysts. Bar graph depicting the absolute SmJNK transcript levels presented as copy number per ng of total RNA. Bars represent the standard deviation of the mean of three technical replicates. **(B)** Bar graph depicting the relative SmJNK transcript levels in adult worms three, five, seven, and ten days after electroporation with SmJNK dsRNA. Bars represent the relative values of SmJNK transcripts compared to the untreated control (▪), or to the unspecific control-GFP (▪). The dashed line represents the values of the normalized controls. Data are represented as mean fold-difference (+/− SE). Asterisks represent statistically significant differences. Data were analyzed using Unpaired t-test with Welch’s correction (N=3, *p < 0.05, **p < 0.01, ***p < 0.001). **(C)** SmJNK transcript levels in schistosomula exposed to SmJNK dsRNA before RNASeq experiments. Bar graph depicting relative SmJNK transcripts levels in schistosomula two days after exposure to SmJNK dsRNA (▪) compared to the untreated control (▪). Data are represented as mean fold-differences (+/− SE). Transcript levels were determined by RT-qPCR and data analyzed using the ΔΔCt method (*p < 0.05, N = 2). Unpaired *t* test with Welch’s correction.

The expression profile of SmJNK in developmental stages of *S. mansoni* (cercariae, two and seven days schistosomula, adult males, adult females, and sporocysts) was investigated by quantitative PCR. Absolute quantification was employed to assess SmJNK expression among the different developmental stages. The SmJNK gene exhibited the highest expression levels (∼4,000 copies/ng of total RNA) in two days schistosomula. This was six times the amount presented in adult males that exhibited the second highest expression levels (∼635 copies/ng of total RNA) (**Figure 1A**). Cercariae, sporocysts and seven days transformed schistosomula presented approximately the same amount of SmJNK transcripts.

### SmJNK Knockdown in Adult Schistosomes

**Figure 2:**
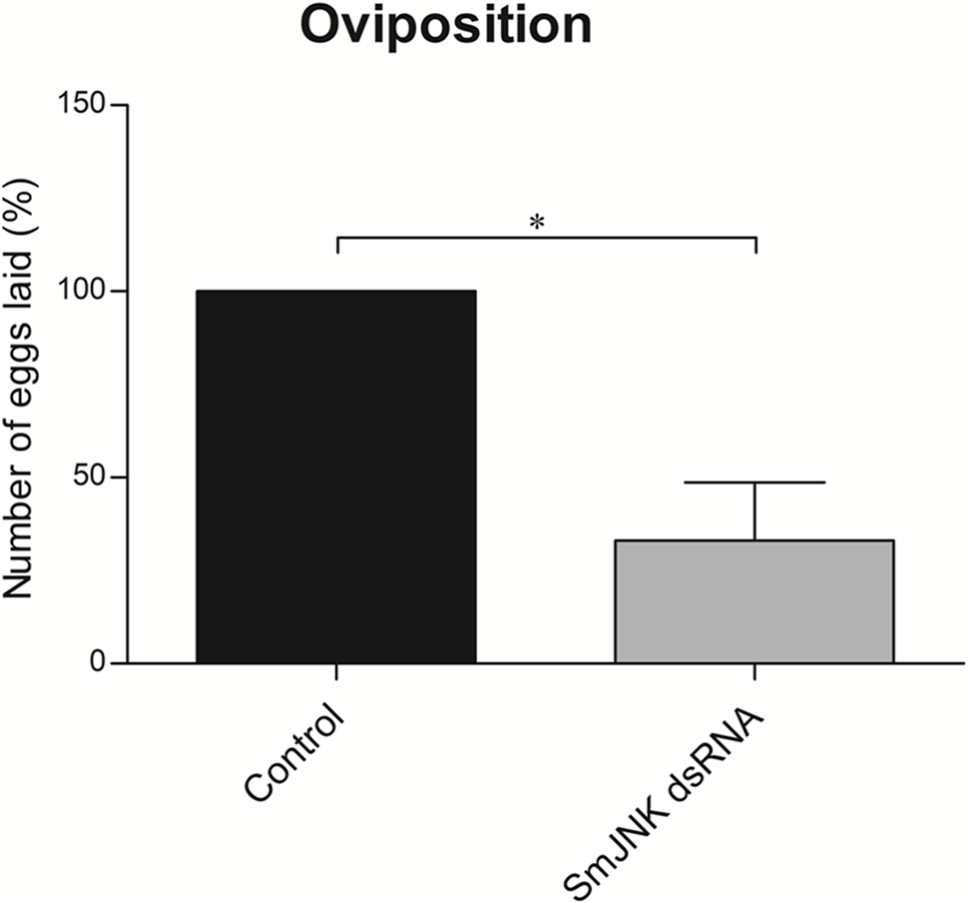
*In vitro* SmJNK knockdown effects in adult schistosome oviposition. Bar graph depicting the percentage of eggs released during 10 days after electroporation with SmJNK dsRNA (▪) compared to untreated control (▪). Data were analyzed using Unpaired t-test with Welch’s correction (N = 3; *p < 0.05).

Adult worms electroporated with SmJNK dsRNA showed up to 62% reduction in SmJNK transcript levels on the fifth day after electroporation (**Figure 1B**). After the successful establishment of SmJNK knockdown in adults, we evaluated phenotypic changes, including worm movement and oviposition. SmJNK knockdown in adult worms did not result in any significant changes in male nor female movement for ten days (**Supplementary Figures S1A, B**). However, a significant reduction of 67% in egg laying was observed in worms electroporated with SmJNK dsRNA (**Figure 2**).

### SmJNK Knockdown in Schistosomula and Quality Assessment of RNASeq Libraries

After confirming successful SmJNK knockdown by RT-qPCR in schistosomula (**Figure 1C**), four paired-end libraries were generated yielding a total of 158 M paired-end reads with 100 bps as average sequence length, with 38–40% guanine-cytosine (GC) content (**Table 1**). FastQC quality assessment indicated that RNASeq data presented a high quality and were adequate for downstream transcriptome analysis. Raw reads were submitted to Sequence Read Archive (SRA; http://www.ncbi.nlm.nih.gov/sra), under accession numbers: PRJNA354932 and PRJNA492452. Sequence mapping to *S. mansoni* reference genome (v. 7) resulted in uniquely mapped reads ranging from 80.94 - 84.16%.

**Table 1:** Summary of sequenced datasets and mapping to the *S. mansoni* reference genome.

Sample	Replicate	Input reads number	Uniquely mapped reads (%)	Reads mapped to multiple loci (%)
Control	1	46650550	80,94	15,35
	2	58555610	82,41	13,86
SmJNK dsRNA	1	29013869	81,31	11,75
	2	23856371	84,16	10,87

**Figure 3:**
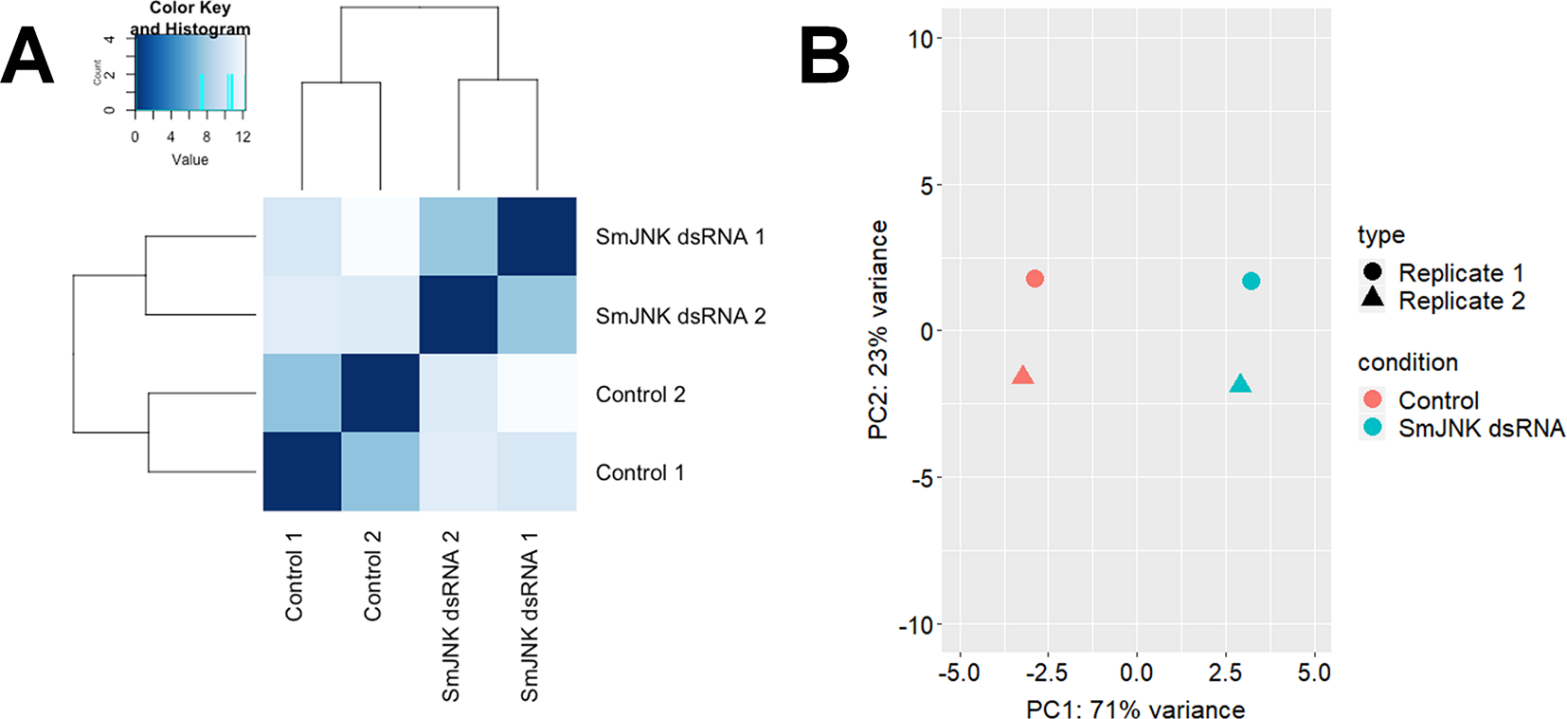
Correlation plots of SmJNK knocked-down schistosomula libraries. **(A)** Heatmap showing the Euclidean distances between the RNASeq libraries of schistosomula treated with SmJNK dsRNA and the untreated control. **(B)** Graph of the first two principal components (PCA) of schistosomula transcriptome expression patterns treated with SmJNK dsRNA compared to the untreated control. The colors represent each treatment: Control (orange) and SmJNK dsRNA (light blue), and the shapes represent each of the two biological replicates (circle and triangle).

The correlation degree between the biological samples was investigated by hierarchical clustering using the heatmap function of the DESeq2 package and means of the Euclidean distances. Clustering analysis grouped libraries from biological replicates in the same branch (**Figure 3A**). Principal component analysis (PCA) was also performed with the genes by multidimensional scaling of the data matrix (**Figure 3B**). The PCA plot of the first two components showed a clear separation between the control and SmJNK dsRNA treated samples in the first dimension.

### Potential SmJNK Regulated Gene Targets Identified in Schistosomula After RNAi

**Figure 4:**
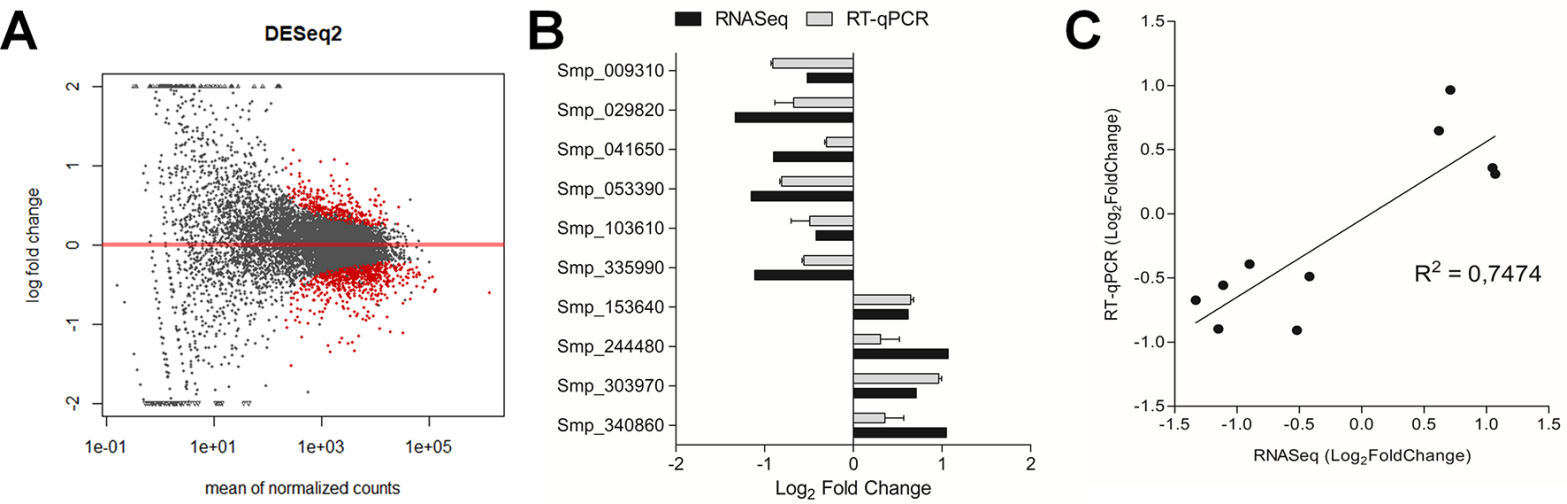
Differentially expressed genes (DEGs) after SmJNK knockdown in schistosomula. **(A)** MA plot depicting a DESeq2 analysis to identify DEGs between SmJNK dsRNA and untreated control. The log-fold change for each transcript is plotted against the mean of normalized counts; each point corresponds to one gene. Significantly altered gene expressions are highlighted in red (padj < 0.01). **(B)** RT-qPCR validation of the differentially expressed genes in response to SmJNK knockdown. Ten DEGs were selected from a range of up-regulated and down-regulated genes. Expression levels were quantified by RT-qPCR (gray) and the results were compared to those obtained by the RNASeq approach (black). **(C)** Pearson’s correlation of Log_2_FoldChange in differentially expressed transcripts between RNASeq and RT-qPCR analysis.

After checking for data consistency and sample quality, the transcriptional profile of schistosomula two days after SmJNK dsRNA exposure was compared to the profile of the untreated control using the DESeq2 package in R. We found 495 DEGs using the DESeq2 package (padj <0.01) (**Figure 4A**). Ten of the DEGs identified among the transcripts were selected for validation by RT-qPCR (**Figure 4B**) (**Supplementary Table S1**). All the analyzed genes showed consistent expression trend between the RNASeq and RT-qPCR analyses, although with some differences in log_2_FoldChange values, which is expected between two different methodologies and among different biological replicates. Significant correlation (R^2^ = 0.7474, p = 0.0012) between the log_2_FoldChange of the employed methodologies confirms the accuracy of the data (**Figure 4C**).

A list of all statistically significant DEGs from schistosomula exposed to SmJNK dsRNA can be found in the **Supplementary Table S2**. Of the 495 DEGs detected in the samples in which SmJNK expression was reduced, 373 are down-regulated and 122 up-regulated in comparison to untreated controls.

### Gene Set Enrichment Analysis

**Figure 5:**
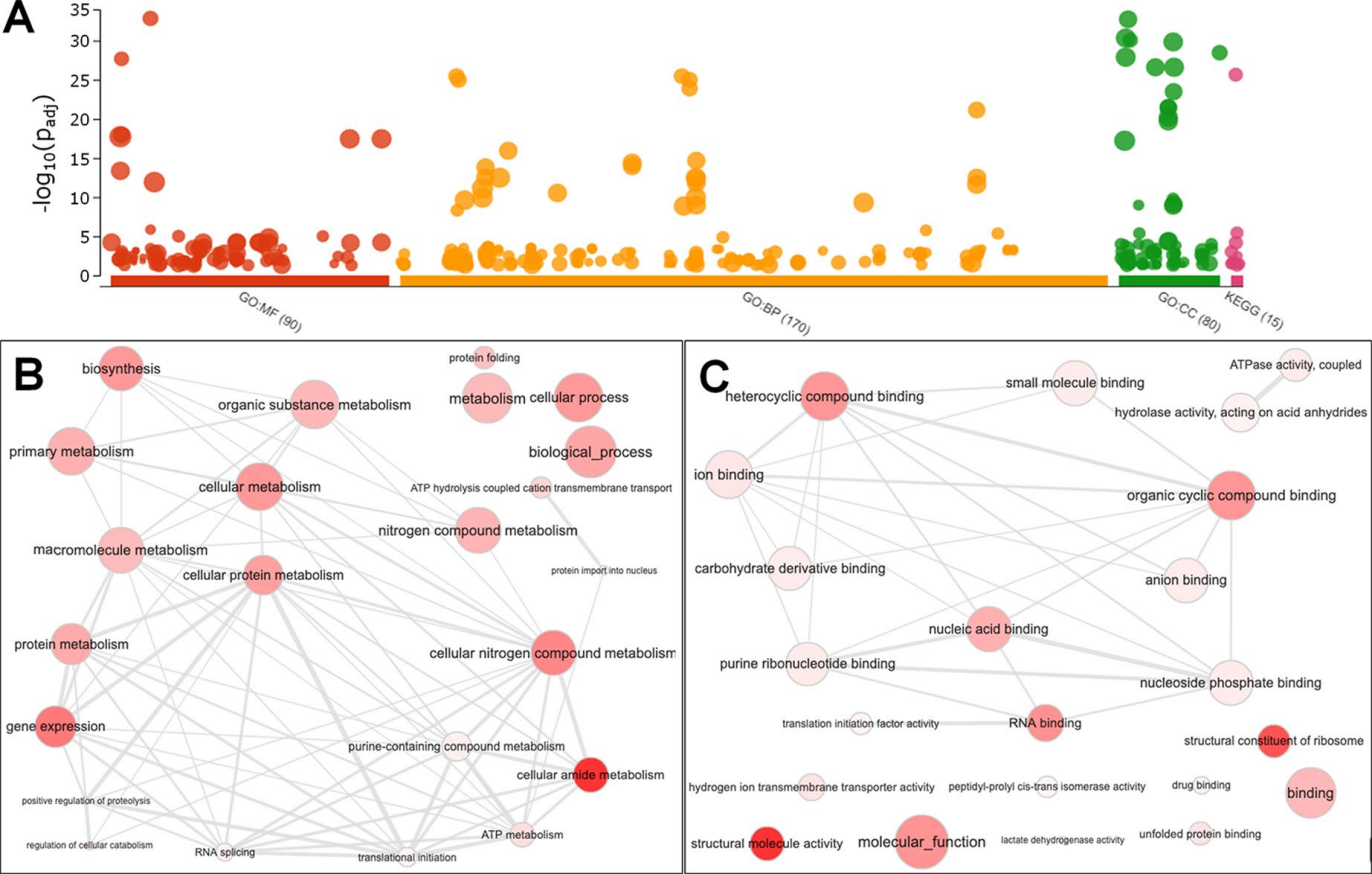
Gene Ontology Enrichment of down-regulated genes from SmJNK knockdown schistosomula. **(A)** Manhattan plot illustrating the enrichment analysis results separated into four categories: GO : MF (Molecular Function), GO : BP (Biological Process), GO : CC (Cellular Component), and KEGG Pathways. The number in the source name in the x-axis labels shows how many significantly enriched terms were found. The circle corresponds to term size. The term location on the x-axis is fixed and terms from the same GO subtree are located closer to each other. Interactive graph of the representative subset of the GO terms related to biological process **(B)** and molecular function **(C)** enriched in down-regulated genes from SmJNK knockdown schistosomula. Bubble colors indicate the p-value and size indicates the frequency of the GO term in the underlying Gene Ontology Annotation database. Highly similar GO terms are linked by edges, where the line width indicates the degree of similarity.

The g:Profiler tool was used to elucidate the functional roles of the 495 DEGs. DEGs were categorized into three GO categories (GO Biological Process, GO Molecular Function, and GO Cell Component) and KEGG Pathways. The significantly enriched subcategories are shown in **Figure 5** and **Supplementary Table S3**. We observed a decrease in expression for genes encoding proteins related to (1) the ribosome structural composition, (2) cytoskeleton-related, (3), purine nucleotide metabolism (4) protein folding, (5) splicing mechanisms, (6) phagosomes, and (7) binding to nucleic acids. For DEGs that showed increased expression, no subcategory showed significant enrichment (**Figure 5A**).

After summarizing the list of GO terms using the ReViGO tool, we found not redundant GO terms and ordered according to the log_10_ p-value. Focusing on enriched GO categories from Biological Process categories found among the down-regulated genes, we found that 23 GOs were representative in SmJNK knockdown schistosomula, due to their uniqueness, we highlight: protein folding, biosynthetic process, adenosine triphosphate (ATP) hydrolysis coupled cation transmembrane transport, ATP metabolic process, regulation of cellular catabolic process, gene expression, nitrogen compound metabolic process, RNA splicing, cellular amide metabolic process, protein import into nucleus, positive regulation of proteolysis, translational initiation, and cellular nitrogen compound metabolic process (**Figure 5B**, **Table 2**). The GO terms included in the molecular function category were summarized in 22 GO terms. We draw attention to structural constituent of ribosome, hydrogen ion transmembrane transporter activity, unfolded protein binding, lactate dehydrogenase activity, peptidyl-prolyl cis-trans isomerase activity, drug binding, translation initiation factor activity, and nucleic acid binding (**Figure 5C**, **Table 2**).

**Table 2:** List of enriched gene ontology (GO) from DEGs down-regulated in schistosomula SmJNK knockdown. The analysis is separated into two GO categories: Biological Process and Molecular Function. The dispensable GO terms are depicted in italic. The values of log_10_p-value refers to log_10_ of padjusted values obtained using g:Profiler toll.

Biological process					
term ID	description	frequency	log_10_ p-value	uniqueness	dispensability
Molecular Function					
GO:0006457	protein folding	0.903%	–72.699	0.93	0.00
GO:0008150	biological_process	100,000%	–102.197	1.00	0.00
GO:0008152	metabolic process	75,387%	–79.533	0.99	0.00
GO:0009058	biosynthetic process	31,611%	–117.905	0.93	0.00
GO:0009987	cellular process	63,780%	–117.165	0.99	0.00
GO:0099132	ATP hydrolysis coupled cation transmembrane transport	0.729%	–46.910	0.79	0.00
GO:0015991	ATP hydrolysis coupled proton transport	0.163%	–14.544	0.77	0.80
GO:0099131	ATP hydrolysis coupled ion transmembrane transport	0.996%	–14.544	0.79	0.96
GO:0009206	purine ribonucleoside triphosphate biosynthetic process	0.485%	–25.175	0.56	0.98
GO:0009145	purine nucleoside triphosphate biosynthetic process	0.485%	–25.175	0.57	0.92
GO:0098662	inorganic cation transmembrane transport	1,858%	–13.551	0.79	0.86
GO:0090662	ATP hydrolysis coupled transmembrane transport	1,001%	–14.544	0.85	0.46
GO:1902600	hydrogen ion transmembrane transport	1,015%	–42.701	0.75	0.82
GO:0006754	ATP biosynthetic process	0.432%	–23.137	0.54	0.97
GO:0015988	energy coupled proton transmembrane transport, against electrochemical gradient	0.170%	–14.544	0.78	0.69
GO:0015985	energy coupled proton transport, down electrochemical gradient	0.411%	–23.137	0.76	0.88
GO:0015986	ATP synthesis coupled proton transport	0.411%	–23.137	0.42	0.88
GO:0009201	ribonucleoside triphosphate biosynthetic process	0.577%	–22.206	0.56	0.95
GO:0046034	ATP metabolic process	1,263%	–37.694	0.54	0.04
GO:0009205	purine ribonucleoside triphosphate metabolic process	1,366%	–25.175	0.57	0.97
GO:0009259	ribonucleotide metabolic process	2,752%	–18.803	0.58	0.92
GO:0019693	ribose phosphate metabolic process	3,032%	–18.803	0.66	0.67
GO:0006163	purine nucleotide metabolic process	2,435%	–18.803	0.55	0.94
GO:0009144	purine nucleoside triphosphate metabolic process	1,401%	–25.175	0.57	0.94
GO:0009142	nucleoside triphosphate biosynthetic process	0.651%	–22.206	0.57	0.86
GO:0009141	nucleoside triphosphate metabolic process	1,605%	–19.590	0.61	0.71
GO:0009150	purine ribonucleotide metabolic process	2,353%	–20.776	0.55	0.89
GO:0009199	ribonucleoside triphosphate metabolic process	1,458%	–22.206	0.57	0.96
GO:0031329	regulation of cellular catabolic process	0.093%	–22.161	0.66	0.05
GO:0009896	positive regulation of catabolic process	0.072%	–20.507	0.69	0.92
GO:1901575	organic substance catabolic process	4,612%	–14.509	0.71	0.60
GO:0009894	regulation of catabolic process	0.146%	–16.724	0.79	0.43
GO:1903364	positive regulation of cellular protein catabolic process	0.038%	–20.507	0.60	0.94
GO:1903362	regulation of cellular protein catabolic process	0.062%	–17.608	0.61	0.91
GO:1903052	positive regulation of proteolysis involved in cellular protein catabolic process	0.034%	–20.507	0.61	0.97
GO:1903050	regulation of proteolysis involved in cellular protein catabolic process	0.056%	–17.608	0.60	0.97
GO:0031331	positive regulation of cellular catabolic process	0.058%	–20.507	0.64	0.97
GO:0051603	proteolysis involved in cellular protein catabolic process	0.759%	–15.585	0.62	0.73
GO:0044257	cellular protein catabolic process	0.772%	–14.580	0.63	0.94
GO:1901800	positive regulation of proteasomal protein catabolic process	0.030%	–20.507	0.61	0.92
GO:0061136	regulation of proteasomal protein catabolic process	0.048%	–17.608	0.60	0.95
GO:0045732	positive regulation of protein catabolic process	0.054%	–20.507	0.62	0.90
GO:0010467	gene expression	19,671%	–154.664	0.81	0.07
GO:0044260	cellular macromolecule metabolic process	34276%	–79.682	0.75	0.42
GO:0044237	cellular metabolic process	53,061%	–116.849	0.87	0.08
GO:0006807	nitrogen compound metabolic process	38,744%	–86.404	0.93	0.09
GO:0044238	primary metabolic process	53,743%	–90.300	0.92	0.11
GO:0071704	organic substance metabolic process	58,357%	–83.098	0.92	0.12
GO:0043170	macromolecule metabolic process	39,491%	–78.359	0.85	0.18
GO:0008380	RNA splicing	0.413%	–16.333	0.77	0.20
GO:0043603	cellular amide metabolic process	6,879%	–235.632	0.78	0.20
GO:0006606	protein import into nucleus	0.102%	–15.977	0.82	0.24
GO:0034504	protein localization to nucleus	0.129%	–15.977	0.88	0.75
GO:0051170	nuclear import	0.107%	–13.547	0.87	0.88
GO:0045862	positive regulation of proteolysis	0.078%	–20.507	0.69	0.27
GO:0006413	translational initiation	0.518%	–13.840	0.65	0.29
GO:0006412	translation	5,686%	–258.077	0.55	0.66
GO:0006518	peptide metabolic process	5,961%	–248.427	0.67	0.88
GO:0043604	amide biosynthetic process	6,374%	–247.598	0.67	0.89
GO:0043043	peptide biosynthetic process	5,770%	–255.362	0.64	0.93
GO:0034641	cellular nitrogen compound metabolic process	34,137%	–138.052	0.74	0.31
GO:1901576	organic substance biosynthetic process	30,365%	–117.012	0.74	0.59
GO:1901564	organonitrogen compound metabolic process	17,886%	–107.455	0.77	0.42
GO:0009059	macromolecule biosynthetic process	19,548%	–131.642	0.72	0.50
GO:0044249	cellular biosynthetic process	30,048%	–115.243	0.73	0.66
GO:0044271	cellular nitrogen compound biosynthetic process	22,502%	–141.400	0.68	0.45
GO:0034645	cellular macromolecule biosynthetic process	19,291%	–133.236	0.68	0.54
GO:0019538	protein metabolic process	18,489%	–96.161	0.80	0.34
GO:0072521	purine-containing compound metabolic process	2,673%	–17.982	0.73	0.35
GO:1901566	organonitrogen compound biosynthetic process	14,064%	–207.359	0.66	0.51
GO:0044267	cellular protein metabolic process	14,293%	–109.414	0.72	0.38
GO:0003674	molecular_function	100,000%	–174.435	1.00	0.00
GO:0003735	structural constituent of ribosome	2,679%	–276.945	0.90	0.00
GO:0005201	extracellular matrix structural constituent	0.028%	–52.190	0.91	0.56
GO:0005200	structural constituent of cytoskeleton	0.079%	–23.341	0.90	0.61
GO:0005198	structural molecule activity	3,268%	–331.562	0.95	0.00
GO:0005488	binding	55,656%	–113.354	0.98	0.00
GO:0015078	hydrogen ion transmembrane transporter activity	0.926%	–42.870	0.80	0.00
GO:0022853	active ion transmembrane transporter activity	0.923%	–17.206	0.80	0.68
GO:0044769	ATPase activity, coupled to transmembrane movement of ions, rotational mechanism	0.363%	–14.218	0.63	0.89
GO:0046933	proton-transporting ATP synthase activity, rotational mechanism	0.325%	–23.533	0.62	0.81
GO:0019829	cation-transporting ATPase activity	0.609%	–17.206	0.61	0.94
GO:0042625	ATPase coupled ion transmembrane transporter activity	0.853%	–17.206	0.60	0.88
GO:0042623	ATPase activity, coupled	2,501%	–32.190	0.67	0.00
GO:0016462	pyrophosphatase activity	7,130%	–24.489	0.66	0.92
GO:0016818	hydrolase activity, acting on acid anhydrides, in phosphorus-containing anhydrides	7,190%	–23.585	0.66	0.92
GO:0036402	proteasome-activating ATPase activity	0.005%	–22.947	0.77	0.43
GO:0017111	nucleoside-triphosphatase activity	6774%	–26.352	0.66	0.85
GO:0016887	ATPase activity	4,560%	–18.342	0.67	0.74
GO:0051082	unfolded protein binding	0.486%	–42.881	0.89	0.00
GO:0004457	lactate dehydrogenase activity	0.034%	–19.668	0.92	0.02
GO:0004459	L-lactate dehydrogenase activity	0.018%	–19.668	0.92	0.46
GO:0003755	peptidyl-prolyl cis-trans isomerase activity	0.359%	–15.208	0.91	0.03
GO:0016859	cis-trans isomerase activity	0.364%	–15.208	0.91	0.63
GO:0008144	drug binding	0.178%	–15.902	0.90	0.04
GO:0003743	translation initiation factor activity	0.411%	–18.402	0.85	0.05
GO:1901363	heterocyclic compound binding	41,115%	–170.093	0.81	0.08
GO:0097367	carbohydrate derivative binding	17,252%	–31.349	0.84	0.20
GO:0043168	anion binding	20,942%	–31.876	0.81	0.21
GO:0003676	nucleic acid binding	21,226%	–128.362	0.77	0.21
GO:0036094	small molecule binding	21,337%	–33.401	0.83	0.21
GO:0043167	ion binding	33,492%	–40.525	0.82	0.26
GO:0003723	RNA binding	5,283%	–177.913	0.81	0.29
GO:0097159	organic cyclic compound binding	41,137%	–169.872	0.81	0.29
GO:0032555	purine ribonucleotide binding	16,057%	–34.104	0.64	0.31
GO:0017076	purine nucleotide binding	16,107%	–33.609	0.66	0.70
GO:0030554	adenyl nucleotide binding	14,356%	–15.611	0.65	0.81
GO:0035639	purine ribonucleoside triphosphate binding	15,815%	–32.542	0.65	0.70
GO:0005524	ATP binding	14,125%	–14.318	0.64	0.81
GO:0000166	nucleotide binding	20,185%	–32.657	0.65	0.75
GO:0032559	adenyl ribonucleotide binding	14,309%	–15.755	0.65	0.85
GO:0032553	ribonucleotide binding	16,747%	–33.282	0.65	0.80
GO:1901265	nucleoside phosphate binding	20,185%	–32.657	0.77	0.33
GO:0016817	hydrolase activity, acting on acid anhydrides	7,223%	–22.918	0.80	0.37

### Crosstalk Between SmJNK and Smp38 MAPK Pathways

**Figure 6:**
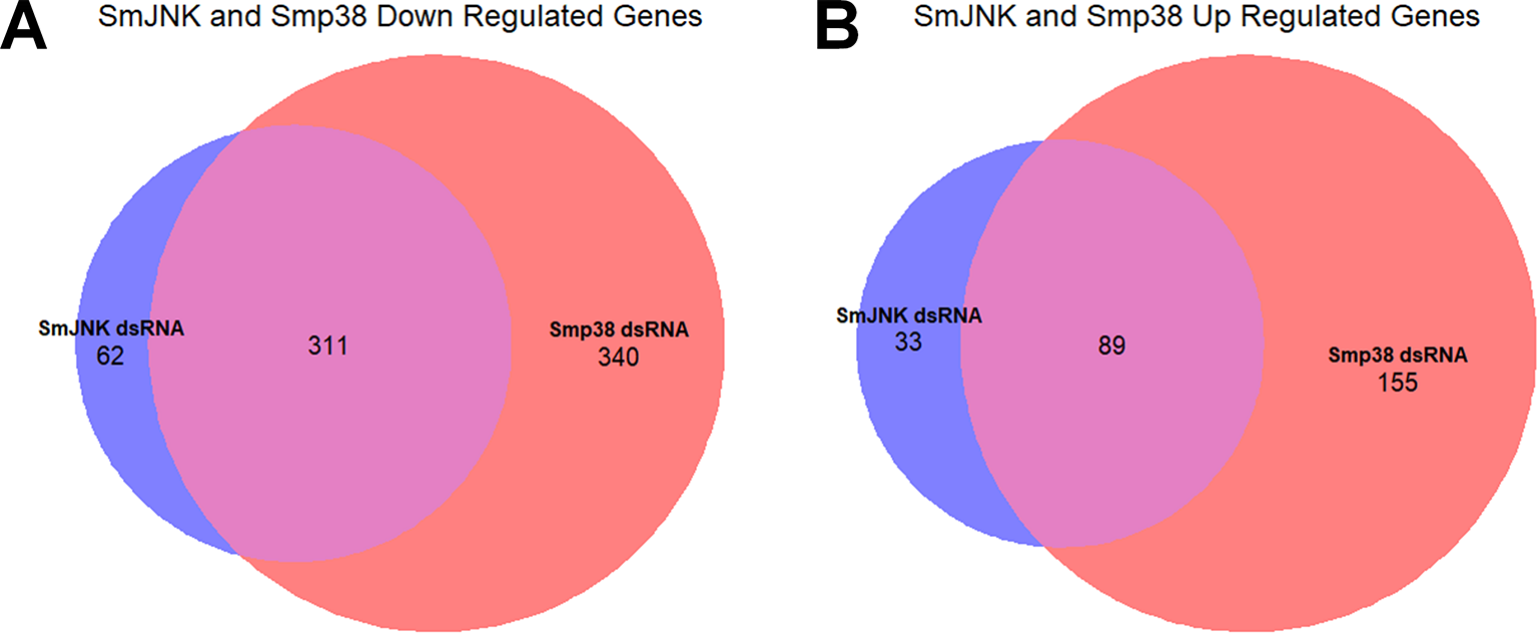
Overlap of DEGs in schistosomula knocked-down for SmJNK and Smp38 MAPKs. Venn diagram representing the number of genes with a decrease **(A)** or increase (B) in gene expression after exposure to SmJNK or Smp38 dsRNAs. In red are represented DEGs regulated by Smp38, in blue SmJNK, and in pink are the overlapping DEGs in both datasets.

Since the JNK and p38 MAPK signaling pathways are triggered by multiple stimuli that simultaneously activate both pathways and consequently share several upstream regulators (Wagner and Nebreda, 2009), we compared the DEGs found here with those found for Smp38 knocked-down schistosomula (Avelar et al., 2019). The SmJNK and Smp38 MAPK pathways in *S. mansoni* presented an intersection of 311 and 89 genes with decreased and increased expression, respectively (**Figure 6**).

**Figure 7:**
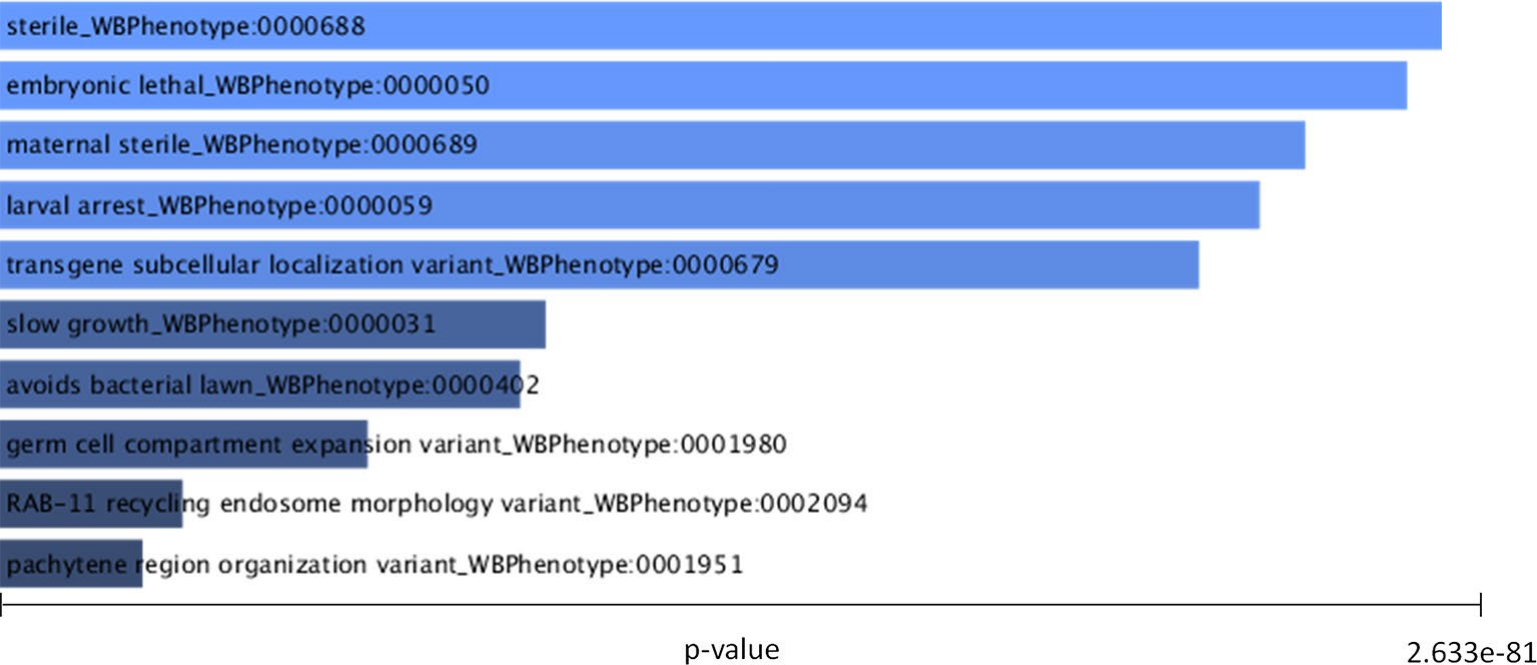
C*. elegans* RNAi phenotypes enriched in orthologues from down-regulated genes in SmJNK and Smp38 knockdown schistosomula. Worm Enrichr analysis was performed to identify the most significantly enriched RNAi phenotypes listed based on the P-value. The length of the bar represents the significance of that specific term. In addition, the brighter the color, the more significant that term is.

Of the 311 down-regulated genes identified in common for SmJNK and Smp38 knockdown schistosomula, we found orthologs for 221 of them, which correspond to 291 different genes in *C. elegans*, since some of the genes present more than one ortholog. The *C. elegans* orthologs are enriched in genes related to the RNAi phenotypes as described: sterile, embryonic lethal, maternal sterile, larval arrest, transgene subcellular localization variant, slow growth, among others (**Figure 7** and **Supplementary Table S4**).

## Discussion

We advanced the understanding of SmJNK gene function by demonstrating that SmJNK is involved in parasite reproduction *in vitro*. We observed a significantly lower number of eggs recovered from SmJNK knocked-down adult worms compared to the control group. It was previously shown that SmJNK knockdown decreased parasite survival and maturation within the definitive host, and that female worms treated with SmJNK dsRNA showed undifferentiated oocytes (Andrade et al., 2014). SmJNK is highly expressed in the ovary of paired females (Lu et al., 2016; Lu et al., 2017), evidencing its role in female reproduction. Therefore, in *in vitro* adult females, SmJNK knockdown may influence oocyte maturation, since the ovary is already fully formed prior to the SmJNK knockdown. This is particularly promising as the eggs are fundamentally related to the disease pathology. Granulomas are the result of host immune response to schistosome eggs. Its formation is triggered by immune cells migration to encapsulate the eggs, resulting in damage of liver tissue (fibrosis and, ultimately, cirrhosis) (Espírito Santo et al., 2008; King and Dangerfield-Cha, 2008; Burke et al., 2009). Additionally, in *in vitro* cultured adult worms, SmJNK does not seem to have a role in parasite movement or viability.

Protein kinases domains are highly conserved between organisms (Manning et al., 2002). Therefore, any drug development effort needs to consider cross-binding of a small molecules that could result in undesired potential side effects. Inhibitors need to be specific and to target essential proteins. The elucidation of gene targets regulated by SmJNK, in addition to contributing to a better understanding of the JNK signaling pathway in *S. mansoni* parasite, also aims at searching for parasite-specific targets. Among the identified DEGs are some proteins with unknown function, these genes could be schistosome-specific. Such proteins may be related to the previously observed phenotypes of parasite development and oviposition impairment.

We observed the down-regulation of genes related to the structural composition of ribosomes in SmJNK knockdown parasites. Ribosomal proteins are major components of ribosomes and play critical roles in protein biosynthesis. Variations observed in the expression of ribosomal proteins in different human tissues are probably related to extraribosomal functions (Bortoluzzi et al., 2001), since ribosomal proteins and RNAs are typically synthesized in stoichiometric amounts (Mager, 1988). Also, the differential expression of ribosomal proteins has been reported in several pathological (Bévort and Leffers, 2000) and stress conditions (Wang et al., 2013).

Genes related to the cytoskeleton showed decreased expression when SmJNK was knocked-down. Integrin-linked kinase (ILK) an activator of the SmJNK, extracellular signal-regulated kinase (SmERK), and Akt pathways, is involved in cytoskeletal reorganization and cell survival. The dysregulation of SmILK may contribute to errors in cell division and genomic instability (Fielding and Dedhar, 2009). The SmILK signaling pathway has an influence on egg production, ovarian structure, and oocyte integrity in female schistosomes (Gelmedin et al., 2017), with similar phenotypes to those observed for SmJNK knockdown (Andrade et al., 2014).

Among genes with decreased expression after SmJNK knockdown, we observed genes related to protein folding, such as chaperonins and heat shock proteins (HSPs). Cellular chaperones such as HSPs confer resistance to stress, promoting cell survival. Prolonged oxidative stress causes an increase in misfolded proteins that aggregate in the endoplasmic reticulum. The objective of the endoplasmic reticulum stress response is to inhibit or retard protein synthesis. This is achieved by the phosphorylation of eukaryotic translation initiation factor 2 (eIF2a) by protein kinase R–like endoplasmic reticulum kinase or by JNK (Nagiah et al., 2016). SmJNK knockdown may have inhibited this stress response, resulting in the observed down-regulation of chaperone and HSPs gene expression.

In rat fibroblasts, positive regulation of the p38 and JNK signaling pathways promotes increased expression of the plasminogen activator inhibitor 1 (PAI-1) (Nutter et al., 2015). JNK modulates positively the PAI-1 expression in the neurodegenerative amyloid pathology observed in Alzheimer’s disease (Gerenu et al., 2017), whereas JNK inhibition significantly attenuates the induction of PAI-1 (Eurlings et al., 2017). PAI-1 was not detected among DEGs in SmJNK and Smp38 knockdown schistosomula. However, the expression of plasminogen activator inhibitor 1 RNA-binding protein (SERBP1, Smp_009310), which plays a role in regulating the PAI-1 mRNA stability, was down-regulated (**Figure 4B**). *Schistosoma* inhibits host coagulation during infection through stimulation of fibrinolytic pathways (Mebius et al., 2013). The interaction between parasites and the hosts’ fibrinolytic system results in the regulation of several functions related to parasite survival mechanisms. For example, the degradation of immunoglobulins and complement components, the activation of matrix metalloproteinases, the stimulation of adhesion, and the degradation of proteins for nutrition (González-Miguel et al., 2016). The detected reduction in the SERBP1 transcript levels, could influence PAI-1 stability, therefore, may interfere in parasite feeding and/or reduce mobility in the host, thus, be related to the parasite survival impairment (Andrade et al., 2014).

SmJNK knockdown also down-regulated genes involved in RNA splicing. Alternative splicing events alter the protein repertoire of the cells being regulated by the expression patterns of the splicing factors (Grosso et al., 2008). The splicing control is as complex and relevant as the transcriptional control (Kornblihtt et al., 2004). It has already been shown that JNK signaling pathway controls splicing events in human T cells (Martinez et al., 2015). This pathway also seems to act in the regulation of alternative splicing in response to extracellular stimuli, promoting changes in splicing patterns (Pelisch et al., 2005). Moreover, *Schistosoma* presents trans-splicing, which is a peculiar mechanism that shares the proteins from the spliceosome, could affect up to 58% of transcripts in the cercarial stage (Boroni et al., 2018). It has been suggested as a target for parasite impairment and would be affected by the alterations detected in categories related to RNA splicing (Mourão et al., 2013).

The ability to deal with adverse environmental conditions is a fundamental need for all organisms, especially parasites. It is, therefore, not surprising that stress-activated protein kinase pathways are among the oldest and most conserved metazoan signaling modules (Twumasi-Boateng et al., 2012). The JNK and p38 MAPK pathways share several upstream regulators and, consequently, multiple stimuli simultaneously activate both pathways (Wagner and Nebreda, 2009). Several studies demonstrate that the extracellular signal-regulated kinase (ERK), JNK, and p38 MAPK signaling pathways act cooperatively, amplifying and integrating signals from various stimuli; promoting appropriate physiological responses including cell proliferation, differentiation, development, inflammatory responses, and apoptosis in mammalian cells (Zhang et al., 2002).

Orthologs of SmJNK and Smp38 in *C. elegans* regulate phenotypes that result in sterile nematodes. Hence, oocyte morphology alterations, decreased number or lack of this structure, and gonad alterations are redundant terms among the enriched RNAi phenotypes in the orthologs. The JNK (named kgb-1) deletion in *C. elegans* has a sterile phenotype, characterized by the massive presence of immature oocytes (Smith et al., 2002). The *C. elegans* kgb-1 deleted mutant presents low reproduction rate and shortened lifespan, concomitantly with reduced expression of genes for protein biosynthesis, chaperones, and enzymes involved in ubiquitination/proteasomal degradation (Gerke et al., 2014). All those findings corroborate the phenotypes and transcription profile associated with SmJNK and Smp38 knockdown.

Here we have shown that SmJNK activity is related to the reduction of egg production *in vitro*. This is in accordance with previously observed phenotype alterations *in vivo*, that demonstrated that SmJNK and Smp38 inhibitors could lead to sterile females, thereby reducing schistosomiasis pathology in the host. Our results point to other key regulatory proteins that are not well conserved between host and parasite, encouraging the development of small-molecule inhibitors.

## Data Availability Statement

The datasets generated for this study can be found in PRJNA354932 and PRJNA492452.

## Ethics Statement

This work was approved by the Oswaldo Cruz Foundation’s Ethics Committee for Animal Use (CEUA) under number LW12/16 , according to the Brazilian national guidelines set out in Law 11794/08.

## Author Contributions

SG, GO, and MM contributed to conception and design of the study. SG and NT performed the experiments. SG performed the bioinformatic and statistical analysis. GO and MM contributed with reagents, materials, and analysis tools. SG, FF, and MM wrote the manuscript. All authors contributed to manuscript revision and read and approved the submitted version.

## Funding

This work has been supported by funding from the European Commission’s Seventh Framework Programme for research, under Grant agreement no. 602080 (AParaDDisE), FAPEMIG (CBB-APQ-0520-13), CNPq grant (302518/2018-5) to MM; and Coordenação de Aperfeiçoamento de Pessoal de Nível Superior–Brasil (CAPES)–Finance Code 001, PCDD Programa CAPES/Nottingham University (003/2014), CNPq grants (470673/2014-1, 309312/2012-4, 304138/2014-2), CAPES (REDE 21/2015), and FAPEMIG (PPM-3500189-13) to GO. SG and NT fellowship was financed by the Coordenação de Aperfeiçoamento de Pessoal de Nível Superior-Brasil (CAPES). The authors thank the support of the Programa de Pós-graduação em Ciências da Saúde, IRR.

## Conflict of Interest

The authors declare that the research was conducted in the absence of any commercial or financial relationships that could be construed as a potential conflict of interest.
