# Supplementary figures and images for: Profiling Transcriptional Regulation and Functional Roles of Schistosoma mansoni c-Jun N-Terminal Kinase
Source: Front Genet. 2019 Oct 18;10:1036. doi: 10.3389/fgene.2019.01036 (PMC6813216; doi:10.3389/fgene.2019.01036)

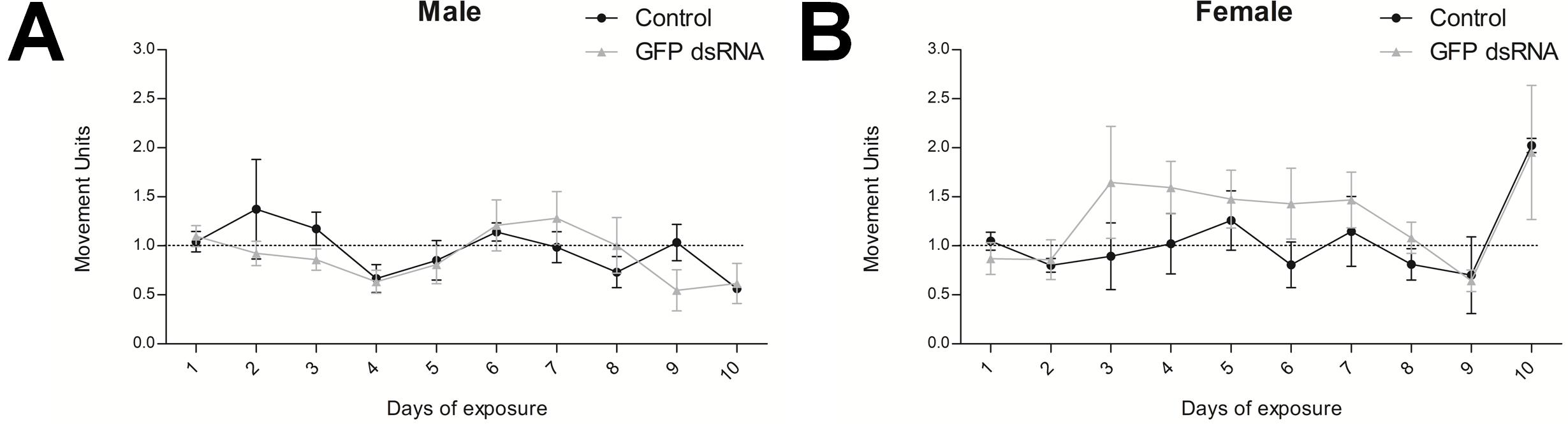

Supplement: Supplementary file 1 [file Image_1.tif]
