# Supplementary material for: Profiling Transcriptional Regulation and Functional Roles of Schistosoma mansoni c-Jun N-Terminal Kinase
Source: Front Genet. 2019 Oct 18;10:1036. doi: 10.3389/fgene.2019.01036 (PMC6813216; doi:10.3389/fgene.2019.01036)
Supplement: Supplementary file 2 [file Presentation_1.pdf]

Supplementary Material

Profiling transcriptional regulation and functional roles of *Schistosoma mansoni* c-Jun N-terminal kinase

Sandra Grossi Gava, Naiara Clemente Tavares, Franco Harald Falcone, Guilherme Oliveira, Marina Moraes Mourão*

* Correspondence:
Marina Moraes Mourão
E-mail: marina.mourao@fiocruz.br

Supplementary Figure Captions

Supplementary Figure S1. *In vitro* SmJNK knockdown effects in adult schistosome movement. Male (A) and female (B) movement units of adult worms knocked-down for SmJNK normalized with untreated (●) or unspecific control (▲). The dotted line represents normalized movement units in the control (N = 3).

Supplementary Tables

Supplementary Table S1. Primer sequences used in RT-qPCR validation of differentially expressed genes.

Gene ID	Gene name	Forward	Reverse	Concentration (nM)	Slope	R^2	Efficiency (%)
Smp_089880	Putative fad oxidoreductase	TACGGTTTCTCTTCCCAACC	CATGGACCTGCTGCATTTAC	400	-3.32	0.996	100.1
Smp_093230	Actin-related protein 10, arp10	GAAGGCTTGGAAGGATTCTG	CAACTGCCAACTCTCGGATA	200	-3.27	0.999	102.2
Smp_153640	PDEase domain-containing protein	CTGGATGGCATGAATTTCTTC	GATGATGTTGACGACGACGA	400	-3.505	0.990	92.9
Smp_244480	putative ataxia telangiectasia mutated (atm)	GAAGAAGAGAAATTACAAGAAACGG	GTATCAGTGAATATAGGCAGAATGG	200	-3.342	0.993	99.2
Smp_303970	Coatomer subunit alpha	TATCACACAGTACCCTCTTCCC	TCAATGTGGACTGTAGTGTAAGC	400	-3.282	0.989	101.7
Smp_340860	Putative 26s proteasome non-ATPase regulatory subunit	TGGATCATTTGACAGTTGTGC	TTTCCCACCATCATCATCAG	400	-3.447	0.994	95.0
Smp_009310	Putative plasminogen activator inhibitor 1 RNA-binding protein	TAGCTCCGACAGAAGCACCT	ACGACCTCGACCAAACATTC	900	-3.37	0.996	98.0
Smp_029820	Putative 60s acidic ribosomal protein P2	AGGCTTGACATCTGCTTGC	CCACAACTGATTGCTGAAGG	1200	-3.383	0.994	97.5
Smp_041650	Putative 40s ribosomal protein S27	AACGTTTGGTTCCCTCACC	TGACATAGAATTCGGTCACATCC	800	-3.389	0.995	97.3
Smp_053390	Histone H4	GCGACGAATAGCAGGCTTAG	AAAGGTGGGAAAGGTCTTGG	400	-3.474	0.993	94.0
Smp_103610	Cathepsin B-like cysteine proteinase	ACTTGGTGGGCACGCTATAC	GGCTGTTACCTCGGATTCAA	200	-3.364	0.996	98.3
Smp_335990	Putative ubiquitin	CTGGTAAGCAGCTAGAAGATGG	GCTCAACTTCCAAAGTGATCG	1200	-3.332	0.984	99.6

Supplementary Table S2. List of statistically significant DEGs from schistosomula exposed to SmJNK dsRNA. Each table contains log2 Fold Change and padj values obtained by DESeq2 package.
EXCEL FILE (TableS2.cvs)

Supplementary Table S3. List of enriched Gene Ontology categories and KEGG Pathways from downregulated genes in SmJNK knockdown schistosomula. The analysis is separated into three GO categories (GO Biological Process, GO Molecular Function, and GO Cellular Component) and KEGG Pathways ($padj < 0.05$).

**Biological Process**					
**term_name**	**term_id**	**adjusted_p_value**	**term_size**	**query_size**	**intersection_size**
translation	GO:0006412	1,56E-26	242	312	51
peptide biosynthetic process	GO:0043043	2,91E-26	245	312	51
peptide metabolic process	GO:0006518	1,44E-25	253	284	49
amide biosynthetic process	GO:0043604	1,74E-25	254	284	49
cellular amide metabolic process	GO:0043603	2,73E-24	269	284	49
organonitrogen compound biosynthetic process	GO:1901566	1,84E-21	427	284	57
cellular nitrogen compound metabolic process	GO:0034641	1,57E-14	1153	371	99
gene expression	GO:0010467	3,42E-16	863	371	86
cellular nitrogen compound biosynthetic process	GO:0044271	7,24E-15	740	361	75
cellular macromolecule biosynthetic process	GO:0034645	4,75E-14	748	361	74
macromolecule biosynthetic process	GO:0009059	6,85E-14	753	361	74
cellular biosynthetic process	GO:0044249	2,99E-12	920	346	78
biosynthetic process	GO:0009058	1,62E-12	950	319	76
cellular process	GO:0009987	1,92E-12	2960	341	166
organic substance biosynthetic process	GO:1901576	1,99E-12	933	319	75
cellular metabolic process	GO:0044237	2,07E-12	2073	371	139
protein metabolic process	GO:0019538	2,42E-10	1133	284	74
biological_process	GO:0008150	6,03E-11	3995	371	214

primary metabolic process	GO:0044238	9,33E-10	2189	371	137
cellular protein metabolic process	GO:0044267	1,14E-11	928	284	68
organonitrogen compound metabolic process	GO:1901564	1,8E-11	1297	284	83
nitrogen compound metabolic process	GO:0006807	2,29E-09	2034	341	121
organic substance metabolic process	GO:0071704	4,9E-09	2260	371	138
cellular macromolecule metabolic process	GO:0044260	1,08E-08	1466	341	95
metabolic process	GO:0008152	1,11E-08	2518	371	151
macromolecule metabolic process	GO:0043170	1,46E-08	1871	341	112
protein folding	GO:0006457	5,37E-08	44	366	14
ATP hydrolysis coupled cation transmembrane transport	GO:0099132	0.00002036949264641862	24	368	9
proton transmembrane transport	GO:1902600	0.00005369077541597394	34	368	10
ATP metabolic process	GO:0046034	0.00017004833961436932	38	368	10
purine nucleoside triphosphate biosynthetic process	GO:0009145	0.003037670897198561	18	316	6
purine nucleoside triphosphate metabolic process	GO:0009144	0.003037670897198561	18	316	6
purine ribonucleoside triphosphate biosynthetic process	GO:0009206	0.003037670897198561	18	316	6
purine ribonucleoside triphosphate metabolic process	GO:0009205	0.003037670897198561	18	316	6
ATP synthesis coupled proton transport	GO:0015986	0.004856734136094929	12	316	5
ATP biosynthetic process	GO:0006754	0.004856734136094929	12	316	5
energy coupled proton transport, down electrochemical gradient	GO:0015985	0.004856734136094929	12	316	5
nucleoside triphosphate biosynthetic process	GO:0009142	0.006017155969374157	20	316	6
ribonucleoside triphosphate metabolic process	GO:0009199	0.006017155969374157	20	316	6
ribonucleoside triphosphate biosynthetic process	GO:0009201	0.006017155969374157	20	316	6
regulation of cellular catabolic process	GO:0031329	0.0060793489282895165	12	331	5
purine ribonucleotide metabolic process	GO:0009150	0.008363094556938505	53	316	9
positive regulation of proteolysis	GO:0045862	0.00889867157110456	7	327	4
positive regulation of protein catabolic process	GO:0045732	0.00889867157110456	7	327	4
positive regulation of proteolysis involved in cellular protein catabolic process	GO:1903052	0.00889867157110456	7	327	4

positive regulation of catabolic process	GO:0009896	0.00889867157110456	7	327	4
positive regulation of cellular protein catabolic process	GO:1903364	0.00889867157110456	7	327	4
positive regulation of proteasomal protein catabolic process	GO:1901800	0.00889867157110456	7	327	4
positive regulation of cellular catabolic process	GO:0031331	0.00889867157110456	7	327	4
nucleoside triphosphate metabolic process	GO:0009141	0.010989660932852382	22	316	6
purine nucleotide metabolic process	GO:0006163	0.01317340598199164	56	316	9
ribose phosphate metabolic process	GO:0019693	0.01317340598199164	56	316	9
ribonucleotide metabolic process	GO:0009259	0.01317340598199164	56	316	9
purine-containing compound metabolic process	GO:0072521	0.015914400720549757	63	357	10
regulation of proteasomal protein catabolic process	GO:0061136	0.01734643067712882	8	327	4
regulation of proteolysis involved in cellular protein catabolic process	GO:1903050	0.01734643067712882	8	327	4
regulation of cellular protein catabolic process	GO:1903362	0.01734643067712882	8	327	4
regulation of catabolic process	GO:0009894	0.021262053480788943	15	331	5
RNA splicing	GO:0008380	0.023264121690808364	59	322	9
protein import into nucleus	GO:0006606	0.02525493684655285	8	360	4
protein localization to nucleus	GO:0034504	0.02525493684655285	8	360	4
proteolysis involved in cellular protein catabolic process	GO:0051603	0.027637528021193663	73	327	10
cellular protein catabolic process	GO:0044257	0.03483520942918839	75	327	10
ATP hydrolysis coupled proton transport	GO:0015991	0.03512230510764226	15	368	5
ATP hydrolysis coupled ion transmembrane transport	GO:0099131	0.03512230510764226	15	368	5
ATP hydrolysis coupled transmembrane transport	GO:0090662	0.03512230510764226	15	368	5
energy coupled proton transmembrane transport, against electrochemical gradient	GO:0015988	0.03512230510764226	15	368	5
organic substance catabolic process	GO:1901575	0.035411496770635834	154	367	16
translational initiation	GO:0006413	0.041303296366220654	39	312	7
inorganic cation transmembrane transport	GO:0098662	0.044142864667227576	85	240	9
import into nucleus	GO:0051170	0.044191369106860286	9	360	4
**Cellular Component**					

term_name	term_id	adjusted_p_value	term_size	query_size	intersection_size
cytoplasm	GO:0005737	7,96E-34	716	327	96
ribosome	GO:0005840	1,09E-30	143	311	44
intracellular	GO:0005622	3,88E-31	1535	367	148
intracellular part	GO:0044424	2,46E-30	1485	367	144
ribonucleoprotein complex	GO:1990904	7,35E-29	206	322	50
cell	GO:0005623	3,29E-28	1725	367	153
cell part	GO:0044464	7,7E-27	1674	367	148
protein-containing complex	GO:0032991	8,53E-27	880	372	104
cytoplasmic part	GO:0044444	1,26E-23	528	315	69
intracellular non-membrane-bounded organelle	GO:0043232	1,65E-21	455	301	60
non-membrane-bounded organelle	GO:0043228	1,65E-21	455	301	60
intracellular organelle	GO:0043229	3,18E-20	1211	364	111
organelle	GO:0043226	1,06E-20	1229	364	111
cellular_component	GO:0005575	3,62E-17	3007	368	189
ribosomal subunit	GO:0044391	9,87E-10	31	148	10
intracellular organelle part	GO:0044446	5,19E-09	691	361	60
small ribosomal subunit	GO:0015935	7,48E-09	15	54	6
organelle part	GO:0044422	9,89E-09	702	361	60
proton-transporting two-sector ATPase complex	GO:0016469	0.000033340414340879555	28	368	9
intracellular membrane-bounded organelle	GO:0043231	0.0003254368151697163	843	372	57
membrane-bounded organelle	GO:0043227	0.00042539893327894784	871	372	58
collagen trimer	GO:0005581	0.0008560587184345816	6	290	4
large ribosomal subunit	GO:0015934	0.000998150601320439	16	358	6
proteasome complex	GO:0000502	0.0010641972132517067	35	327	8
endopeptidase complex	GO:1905369	0.0010641972132517067	35	327	8
proton-transporting ATP synthase complex	GO:0045259	0.0017115059409485824	12	316	5
organelle membrane	GO:0031090	0.0022122525506626275	181	240	15

proton-transporting two-sector ATPase complex, catalytic domain	GO:0033178	0.0035717447354012195	12	368	5
mitochondrial envelope	GO:0005740	0.003847683226486142	65	203	8
peptidase complex	GO:1905368	0.005239484569349109	43	327	8
membrane protein complex	GO:0098796	0.008703524495472636	149	368	16
mitochondrial membrane	GO:0031966	0.00896532966217273	57	194	7
nucleosome	GO:0000786	0.012808504060448294	24	356	6
proton-transporting two-sector ATPase complex, proton-transporting domain	GO:0033177	0.016857577457431303	13	240	4
DNA packaging complex	GO:0044815	0.020648794787145514	26	356	6
protein-DNA complex	GO:0032993	0.020648794787145514	26	356	6
envelope	GO:0031975	0.02213081644347884	83	203	8
organelle envelope	GO:0031967	0.02213081644347884	83	203	8
proton-transporting ATP synthase complex, catalytic core F(1)	GO:0045261	0.026153877468782682	5	316	3
proteasome core complex	GO:0005839	0.03677935880748339	15	251	4
mitochondrial part	GO:0044429	0.03861625090962635	90	203	8
bounding membrane of organelle	GO:0098588	0.04261873163212783	85	275	9

Molecular Function

term_name	term_id	adjusted_p_value	term_size	query_size	intersection_size
structural molecule activity	GO:0005198	6,98E-34	185	152	40
structural constituent of ribosome	GO:0003735	2,02E-28	128	301	40
molecular_function	GO:0003674	3,6E-18	5283	373	279
heterocyclic compound binding	GO:1901363	9,79E-18	1845	370	140
RNA binding	GO:0003723	1,62E-18	352	370	55
organic cyclic compound binding	GO:0097159	1,03E-17	1846	370	140
nucleic acid binding	GO:0003676	1,46E-13	1054	370	91
binding	GO:0005488	4,62E-12	3620	373	203
extracellular matrix structural constituent	GO:0005201	0.000006038829067217214	11	102	5
unfolded protein binding	GO:0051082	0.00005150980774374434	28	366	9

proton transmembrane transporter activity	GO:0015078	0.00005164345770840948	41	316	10
ion binding	GO:0043167	0.00008862333838870891	1509	369	90
purine ribonucleotide binding	GO:0032555	0.00038870566181245704	803	368	55
purine nucleotide binding	GO:0017076	0.0004355852488050613	806	368	55
small molecule binding	GO:0036094	0.00045698611524626543	911	368	60
ribonucleotide binding	GO:0032553	0.0004697202767809331	808	368	55
nucleoside phosphate binding	GO:1901265	0.0005424103025568984	874	368	58
nucleotide binding	GO:0000166	0.0005424103025568984	874	368	58
purine ribonucleoside triphosphate binding	GO:0035639	0.0005568939578934083	792	368	54
ATPase activity, coupled	GO:0042623	0.0006040045842672542	72	340	12
anion binding	GO:0043168	0.00064928849977488	900	368	59
carbohydrate derivative binding	GO:0097367	0.0007329760323595684	820	368	55
nucleoside-triphosphatase activity	GO:0017111	0.0023163089567947285	330	340	27
pyrophosphatase activity	GO:0016462	0.0035569348186957144	338	340	27
hydrolase activity, acting on acid anhydrides, in phosphorus-containing anhydrides	GO:0016818	0.0043804768885977894	342	340	27
proton-transporting ATP synthase activity, rotational mechanism	GO:0046933	0.0044334894191827	7	316	4
structural constituent of cytoskeleton	GO:0005200	0.004633497481722036	16	121	4
proteasome-activating ATPase activity	GO:0036402	0.005073570955465585	7	327	4
hydrolase activity, acting on acid anhydrides	GO:0016817	0.005107551169497762	345	340	27
L-lactate dehydrogenase activity	GO:0004459	0.010793763431696754	2	87	2
lactate dehydrogenase activity	GO:0004457	0.010793763431696754	2	87	2
translation initiation factor activity	GO:0003743	0.014447137370812808	47	321	8
ATPase activity	GO:0016887	0.014647710830029931	98	340	12
ATPase coupled ion transmembrane transporter activity	GO:0042625	0.01902985354987977	17	316	5
active ion transmembrane transporter activity	GO:0022853	0.01902985354987977	17	316	5
cation-transporting ATPase activity	GO:0019829	0.01902985354987977	17	316	5
drug binding	GO:0008144	0.02569201274685189	672	368	43

adenyl ribonucleotide binding	GO:0032559	0.02657446646458657	652	368	42
adenyl nucleotide binding	GO:0030554	0.02747297309130325	653	368	42
peptidyl-prolyl cis-trans isomerase activity	GO:0003755	0.030141544695469886	19	308	5
cis-trans isomerase activity	GO:0016859	0.030141544695469886	19	308	5
ATP binding	GO:0005524	0.036997986471330804	641	368	41
ATPase activity, coupled to transmembrane movement of ions, rotational mechanism	GO:0044769	0.03786242193559343	11	316	4
**KEGG Pathway**					
**term_name**	**term_id**	**adjusted_p_value**	**term_size**	**query_size**	**intersection_size**
Ribosome	KEGG:03010	1,26E-26	83	152	26
Phagosome	KEGG:04145	0.0000040246025285053825	41	368	11
Spliceosome	KEGG:03040	0.00011533198327651202	78	370	13
Oxidative phosphorylation	KEGG:00190	0.002152115462101306	51	368	9
ECM-receptor interaction	KEGG:04512	0.009036501095036616	8	187	3
Pyruvate metabolism	KEGG:00620	0.04343022668549986	18	295	4

Supplementary Table S4. List of enriched RNAi phenotypes in *C. elegans* orthologs from downregulated genes in SmJNK knockdown schistosomula.

Term	Overlap
sterile_WBPhenotype:0000688	137/1370
embryonic lethal_WBPhenotype:0000050	185/3015
maternal sterile_WBPhenotype:0000689	119/1002
larval arrest_WBPhenotype:0000059	141/1606
transgene subcellular localization variant_WBPhenotype:0000679	124/1182
slow growth_WBPhenotype:0000031	130/1858
avoids bacterial lawn_WBPhenotype:0000402	76/484
germ cell compartment expansion variant_WBPhenotype:0001980	70/437
RAB-11 recycling endosome morphology variant_WBPhenotype:0002094	61/353
pachytene region organization variant_WBPhenotype:0001951	55/274
lysosome-related organelle morphology variant_WBPhenotype:0002095	58/337
rachis narrow_WBPhenotype:0001941	55/292
RAB-11 recycling endosome localization variant_WBPhenotype:0002107	51/246
chromosome condensation variant_WBPhenotype:0001361	51/247
pleiotropic defects severe early emb_WBPhenotype:0000270	39/122
transgene expression increased_WBPhenotype:0001236	85/1038
gonad vesiculated_WBPhenotype:0001979	45/213
nuclei small_WBPhenotype:0001792	41/172
sister chromatid segregation defective early emb_WBPhenotype:0000772	34/103
protein aggregation variant_WBPhenotype:0001405	59/503
oocyte number decreased_WBPhenotype:0001944	50/333
oocyte morphology variant_WBPhenotype:0001260	48/325
larval lethal_WBPhenotype:0000054	68/770
locomotion variant_WBPhenotype:0000643	87/1347
apoptosis reduced_WBPhenotype:0000182	41/220
sick_WBPhenotype:0000032	62/651
cytoplasmic processing body variant_WBPhenotype:0001794	44/279
diplotene region organization variant_WBPhenotype:0001950	36/201
oocytes small_WBPhenotype:0001945	29/127
cell membrane organization biogenesis variant_WBPhenotype:0001982	42/344
diplotene absent during oogenesis_WBPhenotype:0001954	28/119
embryonic arrest_WBPhenotype:0000867	31/160
rachis morphology variant_WBPhenotype:0001940	39/300
cell corpse degradation variant_WBPhenotype:0010004	20/44
body elongation defective_WBPhenotype:0000242	20/48
accumulated cell corpses_WBPhenotype:0000241	22/65
pattern of transgene expression variant_WBPhenotype:0000961	62/929
gonad small_WBPhenotype:0001957	24/88
pharyngeal development variant_WBPhenotype:0000707	20/51
transgene expression reduced_WBPhenotype:0001278	55/760
receptor mediated endocytosis defective_WBPhenotype:0001425	54/737
anaphase bridging_WBPhenotype:0001875	21/70

extended life span_WBPhenotype:0000061	47/585
no induction of antimicrobial peptide expression after infection_WBPhenotype:0002460	36/340
germ cell compartment morphology variant_WBPhenotype:0001969	34/304
lethal_WBPhenotype:0000062	45/579
protruding vulva_WBPhenotype:0000697	51/763
pachytene progression during oogenesis variant_WBPhenotype:0001946	20/82
chromosome segregation variant_WBPhenotype:0000773	23/123
apoptosis fails to occur_WBPhenotype:0000184	20/83
no oocytes_WBPhenotype:0000291	28/213
intestinal degeneration_WBPhenotype:0002428	14/33
rachis absent_WBPhenotype:0001942	19/87
growth variant_WBPhenotype:0000030	28/244
germ cell compartment expansion absent_WBPhenotype:0001981	17/65
pathogen resistance increased_WBPhenotype:0001014	15/46
early larval arrest_WBPhenotype:0000055	24/188
nuclear appearance variant_WBPhenotype:0001028	25/225
oocyte septum formation variant_WBPhenotype:0001810	22/171
germ cell compartment anucleate_WBPhenotype:0001971	19/124
oocytes lack nucleus_WBPhenotype:0001956	18/121
germline nuclear positioning variant_WBPhenotype:0001952	25/274
molt defect_WBPhenotype:0000638	24/257
lima bean arrest_WBPhenotype:0000361	45566
reduced brood size_WBPhenotype:0000154	47/1003
cell secretion variant_WBPhenotype:0000258	15/90
accumulated germline cell corpses_WBPhenotype:0001180	22/245
germ cell compartment size variant_WBPhenotype:0001973	19/176
nonsense mRNA accumulation_WBPhenotype:0000508	15/111
mRNA surveillance defective_WBPhenotype:0001538	15/112
organism morphology variant_WBPhenotype:0000535	27/420
body wall muscle myosin organization defective_WBPhenotype:0001569	16/155
apoptosis variant_WBPhenotype:0000730	17/182
shortened life span_WBPhenotype:0001171	26/444
gonad development variant_WBPhenotype:0000691	20/273
slow embryonic development_WBPhenotype:0000042	32112
gonad morphology variant_WBPhenotype:0001355	16/182
multiple nuclei oocyte_WBPhenotype:0001955	28065
excess intestinal cells_WBPhenotype:0001636	12/101
development variant_WBPhenotype:0000518	12997
passage through meiosis defective early emb_WBPhenotype:0000776	24016
cell proliferation increased_WBPhenotype:0000172	12/103
pathogen susceptibility increased_WBPhenotype:0001013	13/134
germ cell compartment multinucleate_WBPhenotype:0001972	15/184
germ cell partition morphology variant_WBPhenotype:0001977	34639
meiotic progression during oogenesis variant_WBPhenotype:0000313	47300
protein expression reduced_WBPhenotype:0000120	16/223
sterile progeny_WBPhenotype:0001037	31/763

foraging behavior variant_WBPhenotype:0000662	13/149
embryonic morphology variant_WBPhenotype:0001136	13332
oocytes disorganized_WBPhenotype:0001925	26177
nuclei enlarged_WBPhenotype:0001567	34608
dauer lifespan extended_WBPhenotype:0002277	28/694
fat content reduced_WBPhenotype:0001183	20/423
paralyzed_WBPhenotype:0000644	11/135
embryonic lethal late emb_WBPhenotype:0001020	27242
variable cell division timing_WBPhenotype:0002390	27242
mitochondria alignment variant_WBPhenotype:0001577	10/125
diakinesis progression during oogenesis variant_WBPhenotype:0001948	28338
germ cell development variant_WBPhenotype:0000812	11/154
general pace of development defective early emb_WBPhenotype:0000126	21367
transgene expression variant_WBPhenotype:0000306	12/190
dead eggs laid_WBPhenotype:0000274	30529
nuclear fallout_WBPhenotype:0001953	22463
germ cell compartment small_WBPhenotype:0001974	23924
germ cell partition absent_WBPhenotype:0001978	42095
increased levels of reactive oxygen species_WBPhenotype:0002238	43619
diplotene progression during oogenesis variant_WBPhenotype:0001947	19146
ATP levels reduced_WBPhenotype:0001575	43191
germ cell morphology variant_WBPhenotype:0000900	28672
patchy coloration_WBPhenotype:0001029	8/106
diakinesis region organization variant_WBPhenotype:0001949	13271
small_WBPhenotype:0000229	15/344
thin_WBPhenotype:0000164	11/207
unfertilized oocytes laid_WBPhenotype:0000357	43711
L1 arrest_WBPhenotype:0000081	22433
motor neuron morphology variant_WBPhenotype:0001331	45383
synapse density variant_WBPhenotype:0002001	45383
exploded through vulva_WBPhenotype:0000038	15/372
mRNA levels increased_WBPhenotype:0000136	33786
life span variant_WBPhenotype:0000039	10/187
cell membrane morphology variant_WBPhenotype:0001399	43802
rachis wide_WBPhenotype:0001943	26451
embryo osmotic integrity defective early emb_WBPhenotype:0000365	8/130
organism development variant_WBPhenotype:0000531	18/527
peptide uptake by intestinal cell decreased_WBPhenotype:0002082	18749
adult lethal_WBPhenotype:0000060	28642
protein expression absent_WBPhenotype:0001410	41699
cell division precocious_WBPhenotype:0001495	41699
organism heat response variant_WBPhenotype:0001273	42430
late larval arrest_WBPhenotype:0000056	31199
clear_WBPhenotype:0001010	10/221
protein transport variant_WBPhenotype:0001864	43525
ectopic expression antibody_WBPhenotype:0001404	43525

protein subcellular localization variant_WBPhenotype:0000436	9/197
male gonad development variant_WBPhenotype:0001675	7/128
embryonic terminal arrest variable emb_WBPhenotype:0000051	43891
mitochondrial content increased_WBPhenotype:0002267	43618
